# Persistence of Cryoglobulinemic Vasculitis after DAA Induced HCV Cure

**DOI:** 10.3390/jcm11040984

**Published:** 2022-02-14

**Authors:** Mahmood Danishwar, Zahid Jamil, Salman Khan, Michael Nakhla, Ishtiaq Ahmad, Muhammad Ashar Ali, Daryl T. Y. Lau

**Affiliations:** Liver Center, Department of Medicine, Beth Israel Deaconess Medical Center, Harvard University, Boston, MA 02115, USA; mahmood.danishwarmd@gmail.com (M.D.); zahidjamil264@gmail.com (Z.J.); salmankhanhoti.khan@gmail.com (S.K.); michael.h.nakhla@gmail.com (M.N.); ishtiaqpwr@gmail.com (I.A.); dlau@bidmc.harvard.edu (D.T.Y.L.)

**Keywords:** cryoglobulinemic vasculitis, DAA therapy, cryoglobulinemia, hepatitis C

## Abstract

Treatment with a direct acting antiviral (DAA) has revolutionized HCV therapy, as more than 95% of patients achieve a sustained virological response (SVR). Cryoglobulinemic vasculitis (CryoVas), however, can persist and recur after the HCV cure. In this systematic review, we include data from 19 studies that provided information on the persistence and recurrence of CryoVas after the HCV cure with DAAs. A complete clinical response (CR) was reported in 63.7% to 90.2% of the DAA-treated patients after achieving SVR. Relapse of CryoVas symptoms was reported in 4% to 18% of the patients. Neuropathy, nephropathy, and dermatological complications were the most common manifestations of CryoVas. B-cell clones persisted in 31–40% of the patients and could contribute to CryoVas relapse. INFL3-rs12979860, ARNTL-rs648122, RETN-rs1423096, and SERPINE1-rs6976053 were associated with a higher incidence of persistence and recurrence of CryoVas. Prospective multicenter studies with diverse patient populations are needed to validate these findings for the timely and effective management of this challenging condition.

## 1. Introduction

**Figure 1:**
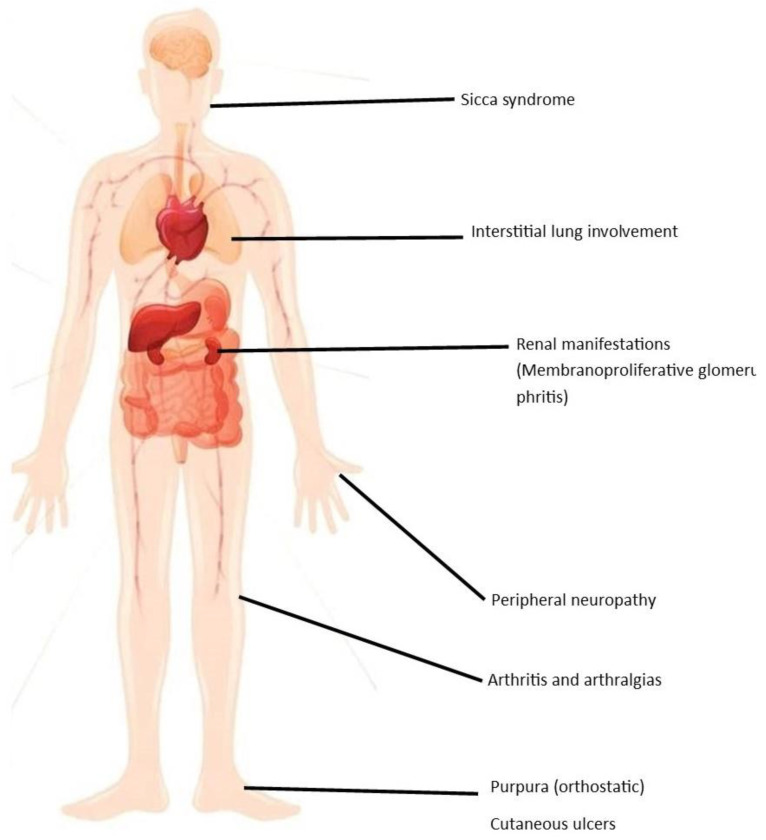
Extrahepatic manifestations of HCV-related cryoglonulinemia.

Hepatitis C virus (HCV) infection is a global health issue affecting more than 180 million people worldwide [1]. It is a major cause of cirrhosis and HCC and is associated with significant extrahepatic manifestations, including autoimmune or lymphoproliferative disorders, and cardiovascular, renal, dermatological, metabolic, and central nervous system diseases (Figure 1) [2].

HCV-related mixed cryoglobulinemia accounts for more than 90% of the cryoglobulinemic vasculitis (CryoVas) [3,4]. Circulating cryoglobulins are present in 40–60% of patients with chronic hepatitis C (CHC); among those, approximately 10% have the symptomatic disease [5].

HCV antigens can exert chronic stimulus on the immune system of susceptive hosts, which can lead to the production of a variety of autoantibodies, such as rheumatoid factor (RF), as well as cryo and non-cryoprecipitate immune complexes (ICs) [6]. Type I cryoglobulinemia (CG) has only one subclass of immunoglobulin (mainly IgG or IgA or IgM) and is primarily associated with lymphoproliferative disorders, such as Multiple Myeloma, Waldenstrom’s macroglobulinemia, chronic lymphocytic leukemia, and B-cell non-Hodgkin’s lymphoma.

Type II mixed cryoglobulinemia comprises a monoclonal IgM with RF activity and a polyclonal Ig (mainly IgG). It is mainly associated with HCV. Type III mixed cryoglobulinemia has polyclonal mixed Ig with RF activity of one polyclonal component (usually IgM). It is mainly found in autoimmune disorders, and less frequently with HCV. Lastly, Type II and Type III mixed cryoglobulinemia can exist together as Type II—III mixed cryoglobulinemia [6].

Mixed cryoglobulinemia manifestations range from asymptomatic circulating cryoglobulins to a cryoglobulinemic syndrome affecting multiple organs [7,8]. The most common presenting features are purpura, arthralgia, polyneuropathy, membranoproliferative glomerulonephritis type 1, B-cell lymphoma, endocrinopathies, and widespread vasculitis [8,9].

The development of direct-acting antivirals (DAAs) marked the new era of HCV therapy; viral clearance with SVR leads to clinical remission of HCV-induced cryoglobulinemia in the majority of patients [10,11]. This review aims to evaluate the prevalence, genetic and risk factors of persistence, or relapse HCV-related CryoVas after a DAA-induced HCV cure.

## 2. Materials and Methods

In conducting this systematic review and meta-analysis, we followed the guidelines established by PRISMA-P [12].

### 2.1. Literature Search

A comprehensive search of several databases with inception to 20 May 2021 was performed using the MeSH “Hepatitis C”, “Cryoglobulinemia”, and “Antivirals”. The databases included PubMed, Embase, Web of Science, Cochrane Library, and Clinicaltrials.gov. The search strategy was designed and conducted as per the PICO framework [13] (Table S1).

### 2.2. Inclusion and Exclusion Criteria

We included all clinical trials and observational studies that provided data in clinical terms about prevalence, complications, predictive factors, and genetics of cryoglobulinemia after treatment of hepatitis C with direct-acting antivirals (DAA). We excluded all case reports, preclinical studies, meta-analyses, review articles, duplicated studies, and clinical studies irrelevant to the study question.

### 2.3. Study Selection

Five researchers (M.D., Z.J., S.K., M.N. and I.A.) reviewed the articles identified through initial search and screened them based on inclusion and exclusion criteria. Two researchers (M.A.A. and D.T.Y.L.) addressed the differences.

### 2.4. Data Extraction

Five researchers (M.D., Z.J., S.K., M.N. and I.A.) extracted the relevant data based on the inclusion/exclusion criteria. Two researchers (M.A.A. and D.T.Y.L.) addressed the differences.

## 3. Results and Discussions

**Figure 2:**
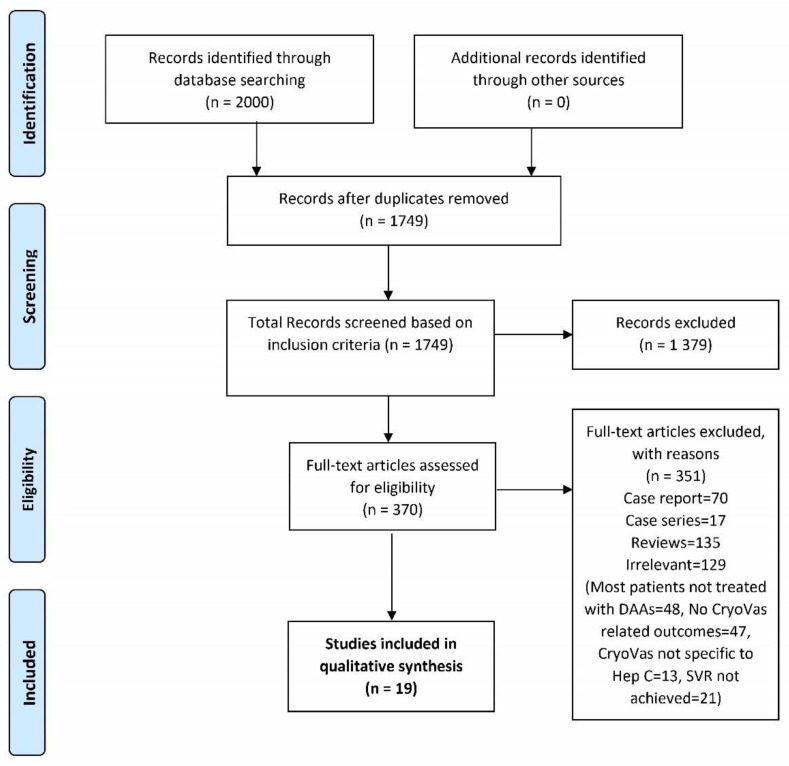
Flow chart of literature search.

Two thousand articles were initially identified through database-searching on PubMed, Embase, Cochrane, Web of Science, and clinicaltrials.gov. Ultimately, 19 studies [14,15,16,17,18,19,20,21,22,23,24,25,26,27,28,29,30,31] meeting the prespecified inclusion criteria were selected (Figure 2).

### 3.1. HCV-Related CryoVas Response with DAA

DAAs are associated with >90–95% SVR rates and have significantly fewer side effects compared to the interferon (IFN)-based regimens [31]. Despite achieving SVR, some patients have persistent circulating cryoglobulins, clinical symptoms, and relapse of CryoVas. The clinical and immunological responses in HCV-induced CryoVas vary greatly depending on the severity of organ involvement, reduction or disappearance of cryoglobulin levels, and genetic and other immune factors, such as C4 and RF activity [18,32].

The complete response (CR) of CryoVas was defined as the improvement of all organ systems along with the absence of clinical relapse. Those with a partial response (PR) had incomplete but >50% improvements in organ systems, whereas patients with non-response (NR) had no improvement or <50% improvement of CryoVas symptoms despite the HCV cure [15]. The definitions were consistent in the included clinical studies. Clinical relapse was reported when there was a recurrence of purpura, arthralgia/arthritis, neuropathy, or nephropathy after CR or PR. Serological relapses were defined by cryoglobulins >1.5%, elevated rheumatoid factor (RF), or detectable complement 4 (C4) after the initial response [33].

Out of the 19 included studies, the CryoVas response and relapse rates were provided in 14 studies and are summarized in Table 1 [14,15,16,17,18,19,20,21,22,23,24,25,26,27]. It includes data from 11 prospective cohort studies, 2 retrospective cohort studies, and 1 cross-sectional study. These studies were from different regions and countries, including five studies from Italy, three from France, three from Egypt, two from Spain, and one International Multicenter study. A total of 1728 patients had CHC treated with DAAs. Overall, a complete response (CR) and partial response (PR) were reported in 63.7–90.2% and 5–50% of the DAA-treated patients, respectively, after achieving SVR. Relapse after CR/PR was mentioned in eight studies and ranged from 0–18% of the treated patients.

Genetics as predictive factors for persistence or relapse CryoVas and persistent B-cell clones were focused on in three [28,29,30] and two studies [26,31], respectively, without providing information about the overall response or relapse. Nephrology complications, neurology complications, and dermatological complications were discussed in four, another four, and six studies, respectively.

#### 3.1.1. Nephrology Complications

Cryoglobulinemic glomerulonephritis refers to the presence of glomerular pathology with circulating cryoglobulins [34]. Cryoglobulinemic vasculitis is characterized by glomerular remodeling due to immune complex deposition, complement activation, and the influx of leucocytes. Cryoglobulinemia is seen in 20% to 29% of the patients with cryoglobulinemia [16,18,35]. The course of the disease is mostly indolent, and the prognosis depends on the severity of the disease. Proteinuria, microscopic hematuria, hypertension, and chronic renal insufficiency are common clinical manifestations of cryoglobulinemic nephritis [36]. Higher SVR rates are achieved with DAA therapy [37]. The prevalence of renal complications after achieving SVR is discussed below.

In a prospective study, Bonicci et al. [18] evaluated 64 DAA-treated HCV patients with circulating cryoglobulins at a single center in Barcelona, Spain. Patients were classified as having asymptomatic circulating cryoglobulins (ACC, n = 29) or symptomatic circulating cryoglobulins (HCV-CV n = 35). Renal involvement was present in seven (20%) of the HCV-CV patients. Overall, 60 patients (94%) had an SVR. Twenty-five patients with HCV-CV (71%) achieved a complete clinical response.

Patients were confirmed to have HCV-CV-related renal disease when at least two of the following parameters were present, in addition to having circulating cryoglobulins: proteinuria > 0.3 g/24 h, hematuria, and a reduced estimated glomerular filtration rate (eGFR) < 60 mL/min/1.73 m^2^, or when cryoglobulinemic membranoproliferative glomerulonephritis was confirmed by a kidney biopsy. A complete renal response was defined by a decrease in proteinuria to <0.3 g/24 h, hematuria resolution, and improvement of at least 20% of eGFR when the baseline value was <60 mL/min/1.73 m^2^.

Renal involvement was present in 7/35 (20%) of the patients with CryoVas. Biopsy-confirmed membranoproliferative glomerulonephritis was present in five of the seven subjects. A complete response was observed in 71% (five of seven) of the patients with renal involvement—a significant improvement in eGFR/proteinuria and disappearance of hematuria. Rituximab was used in three subjects more than 6 months before DAAs, owing to cryoglobulinemic glomerulonephritis (n = 2). However, none of the patients was on rituximab at the time of antiviral therapy. Plasma exchange was performed in one patient with renal involvement more than 12 months before DAAs.

Bonacci et al. [16] evaluated the long-term clinical and immune system effects of HCV eradication with DAAs in 46 patients with a median follow-up duration of 24 (17–41) months. Renal involvement was present in nine patients (20%) and persisted in 3/9 (33%) patients after SVR and even after the prolonged observation.

The VASCUVALDIC 3 study by Cacoub et al. [15] was an international open-label, prospective, multicenter study including 148 patients with active HCV-CryoVas recruited between 2014 and 2017. The primary endpoint was a complete clinical response of CryoVas at week 12 after DAA treatment. Renal improvement was evaluated biologically (proteinuria < 0.3 g/24 h, resolution of hematuria, and improvement of GFR > 20% at week 24 if GFR < 60 mL/min/1.73 m^2^ at diagnosis). The main clinical features of CryoVas at baseline included arthralgia (64.4%), neuropathy (58.1%), purpura (57.4%), glomerulonephritis (16.9%), skin necrosis (10.1%), and other visceral involvement (6.1%). Twelve weeks after DAA discontinuation, 106 (72.6%) patients showed a complete clinical response, 33 (22.6%) showed a partial response, and 7 (4.8%) had no response from their CryoVas manifestations. The majority, 17 of 33 (51.5%) of the partial responders and 3 of 7 (43%) non-responders, had persistent renal insufficiency.

In a cross-sectional Egyptian study by Fayed et al. [21], a total of 12,985 patients with genotype 4 chronic hepatitis C received DAA. All patients had a normal renal function and no signs of immune activation before therapy (normal C4, Negative rheumatoid factor, Negative serum cryoglobulin). They reported 50 cases of de novo renal cryoglobulinemia that developed after successful treatment with DAA. A tissue diagnosis (renal biopsy) showing features of cryoglobulinemia in the presence of seropositivity and significant proteinuria was mandatory for diagnosis. The time interval between the end of DAA treatment and the development of renal affection was 4.3 ± 1.4 months. Renal biopsy of the patients identified the tubulointerstitial disease in all; 26 and 19 also had glomerular and vascular involvement, respectively. De novo renal cryoglobulinemia was treated with methylprednisolone+ plasmapheresis in 26 (52%) patients and methylprednisolone+ cyclophosphamide in 24 (48%). Partial remission with a 50% reduction of proteinuria was achieved in 46%, while complete remission with proteinuria less than 1 g and normal complement levels were achieved in 44%. The de novo renal cryoglobulinemia mechanisms are unclear and need to be confirmed. In this cross-sectional study, only HCV genotype 4 was included. Staining the biopsy tissues with M-Type Phospholipase A2 receptor to differentiate between primary and secondary membranous nephropathy would be very important in future studies.

#### 3.1.2. Neurological Complications

The prevalence of peripheral neuropathy was recently reported to be 50–86% in large series of Mixed Cryoglobulinemic patients [38]. It is mostly associated with type 2 and 3 CG and presents clinically as mononeuropathy, multiple mononeuropathy, or polyneuropathy.

There are different pathogenic mechanisms involved in peripheral neuropathy [39]. It can be due to intravascular deposits of cryoglobulins which interfere with vasa nervorum microcirculation [40]. It can also be a manifestation of vasculitis-induced nerve ischemia. The HCV-associated inflammatory cascade is possible since HCV RNA was detected in the epineural cells. Immune-mediated demyelination leading to axonal degeneration has also been reported [41,42].

In a prospective study, Hassan et al. [17] enrolled 120 treatment-naïve patients with chronic hepatitis C. Among them, 63 patients were tested positive for cryoglobulins with a mean age of 54.2 years. All 63 patients received the sofosbuvir and daclatasvir therapeutic regimen. Peripheral neuropathy, a frequent manifestation of HCV-CryoVas, was present in 33 of the 63 subjects. A total of 28 (84.8%) patients reported a significant improvement in their neuropathic symptoms.

Mazzaro et al. evaluated 22 patients with HCV-related CryoVas. Peripheral neuropathy was present in 10 cases (45%). Improvement in neuropathic pain and paresthesia was observed in seven patients (50%) at the end of treatment, and they remained in complete remission 48 weeks post-HCV therapy. At the end of the 48-week follow-up, no improvement was observed in three cases (33%) with peripheral neuropathy, and they were considered non-responders.

In a prospective, open-label, multicenter study by Saadoun et al. [25], 41 patients with active HCV-associated CryoVas were recruited from hospitals in Paris, France. The median age was 56 years, which included 53.6% women. The patients received sofosbuvir plus daclatasvir for 12 weeks (n = 32) or 24 weeks (n = 9). A total of 21 out of 41 (51.2%) patients had peripheral neuropathy, which included eight patients with sensory-motor polyneuropathy, six with sensory polyneuropathy, and seven with sensory-motor multiplex neuropathy. Peripheral neuropathy improved in 17 out of 21 (81%) cases. Motor symptoms improved in 10 out of 15 patients. The Median Birmingham vasculitis activity (BVAS) score decreased from 8 (4–17) to 0 (0–5). The mean Neuropathy Total Symptom Score-6 (NTSS-6) decreased from 12.4 ± 3.5 to 3.2 ± 2.1. Similarly, Cacoub et al. [15] evaluated 148 patients treated with sofosbuvir-based antiviral therapies for 12 or 24 weeks. Peripheral neuropathy was present in 86 (58.1%) of the study population. A total of 63 (73%) patients had a complete response. Patients with peripheral neuropathy or a severe form of vasculitis presented with skin necrosis, glomerulonephritis, heart, gut, and central nervous system involvement, with two HCV CryoVas conditions that were associated with a poor response to DAA therapy.

#### 3.1.3. Dermatological Complications

Extrahepatic manifestations are well-documented in patients with chronic hepatitis. Studies reported that approximately 74% of HCV-infected patients developed at least one extrahepatic manifestation during their lifetime, and 17% were attributed to dermatological conditions. Mixed cryoglobulinemia, lichen planus, and porphyria cutanea tarda are the most common dermatological complications of HCV infection [24].

Cryoglobulinemic vasculitis is a systemic autoimmune disease classified in the same category as cutaneous leukocytoclastic vasculitis and Henoch–Schonlein purpura. Skin lesions associated with CryoVas usually present as erythematous macules, purpuric papules, and ulcerations in dependent areas, such as the lower extremities. Skin lesions, such as the Raynaud phenomenon, Livedo reticularis, are less frequent [43].

According to an Egyptian study on 1000 patients with chronic hepatitis C by Tawfik et al. [24], 369 patients presented with skin manifestations, and 286 of these 369 (77.5%) patients had cryoglobulinemia. In this and other reports, purpura was the most prevalent skin manifestation that presented in over 50% of patients with HCV-related CryoVas [19,27,28]. Skin necrosis and Raynaud’s phenomenon were reported in 10% and 18% of the patients, respectively [19,27].

In a prospective cohort study, Gragnani et al. observed 17 patients with HCV-CryoVas who achieved a HCV cure with DAA regimens. Positive serum cryocrit was noted in all patients at baseline, along with changes in complement levels and the rheumatoid factor. Among them, 10 were symptomatic, and 7 were asymptomatic prior to DAA therapy. The skin manifestations in the symptomatic group at baseline were purpura in 10, Raynaud’s phenomenon in 2, and ulcers in 3 patients. After 8 weeks of DAA treatment, only two had persistent purpura, and none had ulcers [27]. There was no improvement in Raynaud’s phenomenon. The authors speculated that there could be other etiological factors for Raynaud’s phenomenon in this cohort. Alternatively, a longer follow-up might be necessary to observe the changes.

In another prospective cohort study, Lauletta et al. evaluated 22 patients with HCV-related CryoVas treated with DAAs. The mean baseline cryocrit value was 1.8%, ranging from 0.5% to 4%. Meltzer’s triad of symptoms (purpura, arthralgia, and weakness) was consistently present in all patients. The primary endpoints of the study were (a) SVR at 12 weeks after DAA therapy, (b) a clinical response of CryoVas symptoms, and (c) an immunological response with undetectable cryoglobulin or ≥50% cryocrit reduction. A complete response (CR) was defined as achieving all three primary endpoints; a partial response (PR) was defined as the occurrence of SVR12, with or without either an immunological or clinical response; and no response was defined as the absence of all three endpoints. All patients reached SVR12. CR, PR, and no response at the time of SVR were observed in 14 (63.7%), 5 (22.7%), and 3 (13.6%) patients, respectively [22].

Although clinical improvement is typically reached after achieving SVR, late relapse of HCV-associated CryoVas after DAAs has been reported. Colantuono et al. [20] evaluated 60 patients who responded to DAA therapy. Eleven (18.3%) experienced clinical relapse with purpura and skin ulcers. Visentini et al. [26] and Bonacci et al. [16] reported dermatological relapse in 6.9% and 6.5% of the patients after the HCV cure, respectively.

Overall, DAAs are superior to IFN-based regimens in treating extrahepatic manifestations of chronic hepatitis C, including dermatological conditions [20,22,24,26,27].

#### 3.1.4. Persistent Monoclonal B-Cell Clones after SVR

The hepatitis C virus (HCV) primarily targets the hepatocytes, but it also shows lymphotropism and has the ability to replicate in the lymphocytes [44]. Lymphoid aggregates with the well-formed germinal center are also seen in the liver of chronic HCV-infected patients and suggest an altered environment, with HCV infection favoring retention and proliferation of lymphocytes [45]. HCV can cause monoclonal B-cell lymphoproliferative disorders, including mixed cryoglobulinemia (MC) or non-Hodgkin lymphoma (NHL) [46]. The clonal B-cells have low CD21 expression, or they resemble typical marginal zone B cells. Both types of cells do not respond to BCR or TLR ligands and are considered functionally exhausted [47]. Circulating B-cell clones may persist for some months after HCV eradication, which might be a reason for persistent or relapsed MC. Studies have been conducted on the persistence of B-cells after the eradication of HCV with DAAs.

In the observational study by Schiavinato et al. [31], 29 patients with HCV were treated with DAAs, and all the patients achieved SVR 12. Monoclonal B cells were detected in the blood of nine patients. Five patients were further diagnosed with B-NHL, one with mixed cryoglobulinemia syndrome (MCS), and three had no further diagnostic evaluation. In nine patients with a lymphoproliferative disorder, the global decrease in clonal B-lymphocytes was 39% after HCV eradication. Both B-cell reduction and κ/λ ratio normalization were reported in three patients. Only B-cell reduction was reported in five patients, and no reduction in one patient. In 20 patients without the lymphoproliferative disease, the global decrease in clonal B-cells was 9% after HCV eradication. Monoclonal B-cells were reduced with HCV eradication with DAAs; however, it did not completely eradicate clonal B-cells.

In another observational study by Visentini et al. [26], all 45 patients had HCV-CryoVAS. Eight patients had indolent B-cell NHL, and 18 patients had compensated cirrhosis. All patients achieved SVR after DAA therapy, and were followed-up for a median of 18.5 (9–38) months. After treatment with DAAs, complete response (improvement of all baseline symptoms), partial response (improvement of half of the symptoms), and no response (improvement of less than half of the symptoms) were reported in 35 (78%), 8 (18%), and 2 (4%) of the patients, respectively. A total of 18 (40%) patients had detectable circulating clones of B-cells at baseline, which persisted in 17 patients throughout the follow-up. The median number of clones at baseline was 81% (range: 14–95%) and 96% (range: 78–98%) of the circulating B-cells in CryoVas patients and CryoVas-NHL patients, respectively. However, no correlation was found between CryoVas and B-cell clones, as nine patients had a complete response with no circulating cryoglobulins but still had large detectable clones. The detectable clonal cells might have had functional changes in these nine patients after the clearance of HCV. On the other hand, one patient with high titers of cryoglobulins had complete clearance of B-cell clones. One possible source of cryoglobulins in this patient can be colonal cells confined in the liver or lymphoid tissue [48].

#### 3.1.5. Predictive Factors and Genetics

In the DAA era, persistence or relapse of HCV-CryoVasc after SVR was still present in approximately <5% of patients [49,50,51]. Recent studies suggest the roles of genetic predisposition to the CryoVas response.

In a prospective study by Gragnani et al. [28], 98 patients with HCV-related CryoVac were enrolled after the DAA-induced SVR. Among them, 52 had complete clinical responses, whereas 46 had persistent or relapsed symptoms. Blood samples of the study participants were analyzed for hematologic and genetic markers that correlated with the clinical outcomes following SVR. B-cell clonality markers, the free-light-chain (FLC) k/l ratio, t (14;18) translocation, and monoclonal B lymphocytosis were analyzed. The FLC κ/λ ratio depends on the balance between production and renal clearance of FLC and is altered in many diseases, such as multiple myeloma and monoclonal gammopathy. T (14;18) translocation is the hallmark of follicular lymphoma; it results in overexpression of Bcl2, which inhibits the physiological apoptosis of B cells. In this study, the FLC κ/λ ratio was found to be abnormal in 47% of the patients with persistent or recurrent CryoVac symptoms compared to 17% among complete responders (*p* = 0.003). Similarly, t (14;18) translocation was detected in 40% of non-responders/relapsers and in only 17% of complete responders (*p* = 0.02). Moreover, a high percentage (17%) of monoclonal B lymphocytosis was observed in patients with persistent or relapsed HCV-CryoVasc symptoms vs. 4% in complete responders (*p* = 0.04). Five single nucleotide polymorphisms known to be associated with lymphoproliferative disorders were also examined. Of these, the notch4 rs2071286 single nucleotide polymorphism was present in a significantly higher frequency for the T minor allele (46% vs. 29%, *p* = 0.01; OR, 2.17; 95% CI, 1.18–3.9) and TT genotype (17% vs. 2%, *p* = 0.006) in patients with persistent or relapsed HCV-CryoVac symptoms compared to those with a complete response.

Chang et al. [29] reported the genetic associations with pre-therapy and post-therapy mixed cryoglobulinemia. A total of 1043 HCV antibody-positive Taiwanese patients were prospectively surveyed for mixed cryoglobulinemia. Among the study participants, 56.5% had baseline MC, 89.5% had positive HCV RNA, 796 completed anti-HCV therapy, and 715 achieved SVR. Previously conducted European and US studies have shown the associations of neurogenic locus notch homolog protein 4 (NOTCH4), ATP-binding cassette subfamily B member 1 (ABCB1), and human leukocyte antigen (HLA) class 2 with HCV-associated mixed cryoglobulinemia. The presence of cryoglobulin up-regulates plasminogen activator inhibitor 1 (PAI-1), and PAI-1 inhibits HCV replication. The locus at chromosome 7q22.1 close to Serpin Family E Member 1 (SERPINE1) rs697053 and the aryl hydrocarbon receptor nuclear translocator-like (ARNTL) rs6486122 at chromosome 11p15.2 are highly associated with PAI-1 levels. Additionally, resistin regulates PAI-1 expression via protein kinase B phosphorylation, and resistin (RETN) rs1423096 is associated with resistin levels. The presence of rs697053 and rs1423096 are associated with low PAI-1 levels resulting in a high HCV replication rate. The rs6486122 T allele is associated with high PAI-1 levels, endorsing the concept that the emergence of mixed cryoglobulinemia aids in inhibiting HCV replication. In the multivariate analysis, interferon λ3-rs12979860 C allele and rs6486122 T allele were positively associated with pre-therapy mixed cryoglobulinemia (OR = 1.513; 95% CI = 1.087–2.056, *p* = 0.015 and OR = 1.191; 95% CI = 1.000–1.419, *p* = 0.049 respectively). Like-wise, rs1423096 C allele was associated with 24-week post-therapy mixed cryoglobulinemia (OR = 0.677; 95% CI = 0.46–0.995, *p* = 0.047). rs6976053 T allele was associated with long-term mixed cryoglobulinemia i.e., up to 10-years post-therapy (OR = 0.933; 95% CI = 0.746–1.166, *p* = 0.541).

Hegazy et al. [30] prospectively studied 32 patients with HCV-related CryoVac, 13 patients with HCV infection alone, and 8 healthy controls. Different DAA-based combination therapies were used. The expression of two primary B-cell markers, B-cell activating factor (BAFF) and A proliferation-inducing ligand (APRIL), were measured at multiple points in the study: before, at the end of treatment (EOT), and 6 and 12 months after treatment. The controls were followed for a similar duration. BAFF and APRIL levels continued to rise at follow-up points in patients with CryoVac compared to those with HCV alone. Among the CryoVac patients, BAFF levels peaked at 6 and 12 months after DAA therapy compared to baseline levels (1.91 ± 0.7, *p* < 0.05 and 6.08 ± 2.7, *p* < 0.05, respectively). Similarly, APRIL expression increased to 2.58 ± 0.7 at EOT in HCV-CryoVasc patients, significantly higher than its pre-treatment level (*p* = <0.05). At 6 and 12 months, APRIL levels continued to rise to 2.83 ± 1.4 and 3.77 ± 1.5, respectively (*p* < 0.05). This increased expression of BAFF and APRIL seemed to stimulate B-cell survival and a high chance of relapse of cryoglobulinemia despite initial improvement in cryoglobulinemia and viral clearance.

## 4. Conclusions

Despite achieving SVR with DAA therapy, the persistence of cryoglobulinemia was reported in a significant number of patients. The analysis of hematologic or genetic markers could predict the HCV-CryoVas treatment outcomes. B-cell clonal markers (FLC K/L ratio, t (14;18) translocation and monoclonal B-lymphocytosis) and B-cell activating factors (BAFF and APRIL) were found more frequently in patients with persistent/relapsed HCV-CryoVas symptoms. Among SNPs analysis, INFL3-rs12979860 and ARNTL-rs648122 were more frequently associated with a higher incidence of baseline CryoVas. RETN-rs1423096 and SERPINE1-rs6976053 were associated with a higher incidence of short and long-term post-therapy CryoVas, respectively, in patients with DAA-induced SVR.

Dermatological manifestations were present in nearly all patients with HCV-associated CryoVas, with purpura being the most prevalent among them. The clinical response rate for purpura was high in patients who achieved SVR with DAAs. However, lower clinical response rates were reported for other dermatological manifestations, such as Raynaud’s phenomenon. The membranoproliferative disorder was the most common renal manifestation with CryoVas and can rarely lead to CKD after the HCV cure. DAAs were effective in treating peripheral neuropathy in most patients. However, peripheral neuropathy and a severe form of vasculitis were associated with a relatively poor response to DAA and a higher incidence of relapses compared to other manifestations of HCV CryoVas. Long-term relapses of dermatological manifestations, neuropathy, and glomerulonephritis were also reported.

The HCV cure with DAAs can significantly reduce pathological B-cell clones in the peripheral blood of most patients. However, B-cell clones can persist for a longer duration after HCV and can cause CryoVas relapse with triggers other than HCV.

In future studies of HCV-related CryoVas, hematological and genetic markers should be evaluated to confirm their associations with DAA response and relapse. DAA refractory cryoglobulinemia remains an important unmet need that requires prospective studies and clinical trials to improve our understanding and management of this challenging medical condition.

## Figures and Tables

**Table 1:** CryoVas response to DAA-induced HCV cure and risk of relapse.

Studies	Study Design	Location	Follow Up	No Response	Complete Response (CR)	Partial Response (PR)	Relapse After CR/PR
Mazzaro et al., 2018 [14]	Open label prospective pilot study	Italy (multicenter)	48 weeks SVR	5 (23%)	17 (77%)	0	0
Cacoub et al., 2018 [15]	Prospective observational	International Multicenter	Individually tailored	7 (4.8%)	106 (72.6%)	33 (22.6%)	0
Bonacci et al., 2018 [16]	Prospective observational	Barcelona (Spain)	24 months (17–41 months)	4 (9%)	37 (80%)	5 (11%)	5 (11%)
Hassan et al., 2018 [17]	Prospective observational	Egypt	12–24 weeks	0	55 (87%) clinical, immunological, virological	8 (13%)	0
Bonacci et al., 2017 [18]	Prospective observational	Barcelona (Spain)	12–24 weeks	5 (14%)	25 (71%)	5 (14%)	0
Miailhes et al., 2018 [19]	Retrospective Cohort	France	4 years (2013–2017)	Cryoglobulinemia persisted = 34%	N/A	N/A	N/A
Colantuono et al., 2020 [20]	Retrospective Cohort	Rome (Itlay)	30.5 months (11–51 months)	10 (14%)	52 (74%)	8 (11%)	11 (18%)
Fayed et al., 2018 [21]	Cross-sectional study	Cairo (Egypt)	2 years	N/A	N/A	N/A	N/A
Lauletta et al., 2017 [22]	Prospective observational	Italy (single-center)	12–24 weeks	3 (13.6%)	14 (63.7%)	5 (22.7%)	N/A
Comarmond et al., 2017 [23]	Prospective observational	France	24 weeks	N/A	24 (88.9%)	N/A	N/A
Tawfik et al., 2020 [24]	Cross-sectional	Egypt	3 months	N/A	N/A	80% of patients showed improvement	N/A
Saadoun et al., 2017 [25]	Open label, prospective pilot study	France (Multicenter)	26 months (20–30 months)	N/A	37 (90.2%)	4 (9.8%)	0
Visentini et al., 2018 [26]	Prospective observational	Italy	18.5 (9–38 months)	2 (4%)	35 (78%)	8 (18%)	3 (6%)
Gragnani et al., 2016 [27]	Open label prospective pilot	Italy	8 weeks	2 (20%)	3 (30%)	5 (50%)	NA

*Some columns of the studies exceeded 100%; the % of relapsed patients should be excluded in counting the total subjects.*

## Data Availability

Not applicable.

## Supplementary Materials

The following supporting information can be downloaded at: https://www.mdpi.com/article/10.3390/jcm11040984/s1, Table S1: Key words and search string for this article.
