# Supplementary material for: Persistence of Cryoglobulinemic Vasculitis after DAA Induced HCV Cure
Source: J Clin Med. 2022 Feb 14;11(4):984. doi: 10.3390/jcm11040984 (PMC8878349; doi:10.3390/jcm11040984)
Supplement: Supplementary file 1 [file jcm-11-00984-s001.zip › jcm-1553118-supplementary.pdf]

Table S1. PICO table with keywords and search strings from data basis:

P	I	C	O	S
"Hepatitis C"[Mesh]	"Antiviral Agents"[Mesh]		"Cryoglobulinemia"[Mesh]	
Parenterally-Transmitted Non-A, Non-B Hepatitis<br><br>Parenterally Transmitted Non A, Non B Hepatitis<br><br>PT-NANBH<br><br>Hepatitis, Viral, Non-A, Non-B, Parenterally-Transmitted<br><br>Hepatitis C	Agents, Antiviral<br><br>Antivirals<br><br>Antiviral<br><br>Antiviral Drugs<br><br>Antiviral Drug<br><br>Antiviral Agent		Cryoglobulinemia<br><br>Cryoglobulinemias	

PubMed search string:

((((((("Antiviral Agents"[Mesh]) OR (Agents, Antiviral)) OR (Antivirals)) OR (Antiviral)) OR (Antiviral Drugs)) OR (Antiviral Drug)) OR (Antiviral Agent)) AND (((("Cryoglobulinemia"[Mesh]) OR (Cryoglobulinemias)) OR (Cryoglobulinemia)) AND (((((("Hepatitis C"[Mesh]) OR (Parenterally-Transmitted Non-A, Non-B Hepatitis)) OR (Parenterally Transmitted Non A, Non B Hepatitis)) OR (PT-NANBH)) OR (Hepatitis, Viral, Non-A, Non-B, Parenterally-Transmitted)) OR (Hepatitis C)))

--756

Embase search string:

('hepatitis c'/exp OR 'hepatitis c' OR 'hepatitis c virus infection' OR 'parenterally transmitted non a non b hepatitis') AND ('antivirus agent'/exp OR 'agent, virucidal' OR 'anti viral agent' OR 'antiviral' OR 'antiviral agent' OR 'antiviral agents' OR 'antiviral drug' OR 'antiviral substance' OR 'antivirals' OR 'antivirus agent' OR 'viral inhibitor' OR 'virostatic agent' OR 'virucidal agent' OR 'virucide' OR 'virucide agent' OR 'virus repressor' OR 'virustatic' OR 'virustatic agent') AND ('cryoglobulinemia'/exp OR 'cryoglobulinaemia' OR 'cryoglobulinemia' OR 'cryoimmunoglobulinaemia' OR 'cryoimmunoglobulinemia' OR 'mixed cryoglobulinemia') AND

('clinical study'/exp OR 'clinical data' OR 'clinical studies as topic' OR 'clinical study' OR 'medical trial')

--949

WOS search:

With keywords mentioned above=263

Cochrane search:

With keywords mentioned above: 20

Clinicaltrials.gov search:

Hepatitis C + Cryoglobulinemia=12
